# Modeling the time course of ComX: towards molecular process control for *Bacillus* wild-type cultivations

**DOI:** 10.1186/s13568-021-01306-5

**Published:** 2021-10-29

**Authors:** Chantal Treinen, Olivia Magosch, Mareen Hoffmann, Peter Klausmann, Berit Würtz, Jens Pfannstiel, Kambiz Morabbi Heravi, Lars Lilge, Rudolf Hausmann, Marius Henkel

**Affiliations:** 1 Institute of Food Science and Biotechnology, Department of Bioprocess Engineering (150K), University of Hohenheim, Fruwirthstr. 12, 70599 Stuttgart, Germany; 2 Core Facility Hohenheim, Mass Spectrometry Unit, University of Hohenheim, August-von-Hartmann-Str. 3, 70599 Stuttgart, Germany

**Keywords:** Molecular process control, *Bacillus subtilis*, ComX, Quorum Sensing, Wild-type cultivation, Surfactin lipopeptide

## Abstract

Wild-type cultivations are of invaluable relevance for industrial biotechnology when it comes to the agricultural or food sector. Here, genetic engineering is hardly applicable due to legal barriers and consumer’s demand for GMO-free products. An important pillar for wild-type cultivations displays the genus *Bacillus*. One of the challenges for *Bacillus* cultivations is the global ComX-dependent quorum sensing system. Here, molecular process control can serve as a tool to optimize the production process without genetic engineering. To realize this approach, quantitative knowledge of the mechanism is essential, which, however, is often available only to a limited extent. The presented work provides a case study based on the production of cyclic lipopeptide surfactin, whose expression is in dependence of ComX, using natural producer *B. subtilis* DSM 10^ T^. First, a surfactin reference process with 40 g/L of glucose was performed as batch fermentation in a pilot scale bioreactor system to gain novel insights into kinetic behavior of ComX in relation to surfactin production. Interestingly, the specific surfactin productivity did not increase linearly with ComX activity. The data were then used to derive a mathematic model for the time course of ComX in dependence of existing biomass, biomass growth as well as a putative ComX-specific protease. The newly adapted model was validated and transferred to other batch fermentations, employing 20 and 60 g/L glucose. The applied approach can serve as a model system for molecular process control strategies, which can thus be extended to other quorum sensing dependent wild-type cultivations.

**Supplementary Information:**

The online version contains supplementary material available at 10.1186/s13568-021-01306-5.

## Introduction

The genus *Bacillus* is considered to be an important microorganism for industrial applications (Schallmey et al. 2004), especially when targeting wild-type cultivations. Thereby, the species *Bacillus subtilis* has been often described as “cell factory*”* for the production of secretory proteins (Westers et al. 2004; van Dijl and Hecker 2013; Su et al. 2020) and is known for its role as model organism for Gram-positive bacteria (Harwood 1992). But not only the products are of interest, the organism itself shows potential as a plant growth promoting bacterium (Tiwari et al. 2019) as well as for crop protection (Ongena and Jacques 2008). A decisive advantage is that *B. subtilis* has been granted a GRAS status for the production of enzyme preparations (Sewalt et al. 2016), which simplifies industrial implementation. The industrial use of wild-type organisms in the agriculture or food sector of the European Union is preferred due to customer demand and strict legal requirements for genetically modified organisms (GMOs) (Peters and Sawicka 2007; Hanlon and Sewalt 2021). As genetic engineering is not a suitable option for process improvement in this context, “smart process control*”* (Noll and Henkel 2020) is on a rise. In large-scale industrial processes, especially titer, yield and productivity are of great importance (Liu et al. 2018; Venayak et al. 2015). Therefore, the investigation of diverse optimization strategies is of current research interest. One of the challenges for process control and optimization in *Bacillus* spp. is quorum sensing, which is a bacterial cell to cell communication, in which gene expression is regulated in dependence of the cell density (Bassler 1999). One major actor in the regulatory network is the pheromone ComX (Magnuson et al. 1994), which functions as an autoinducer. ComX is produced as a precursor, which is then posttranslationally modified and secreted by the ComQ prenyltransferase (Bacon Schneider et al. 2002). The sensor kinase ComP recognizes the extracellular ComX at a certain concentration and phosphorylates the ComA response regulator. In the phosphorylated state, ComA ~ P activates among others the multi-regulated promotor P_*srfA*_, which promotes surfactin production (Nakano et al. 1991; Comella and Grossman 2005). This relation can be used to explore a potential for molecular process control, using ComX-dependent expression of surfactin as a model system. In this way, the presented study can contribute to establishing efficient bioprocesses by acting as a reference method for quorum sensing controlled cultivations of *Bacillus*. Microbial surfactants, such as surfactin, exhibit a wide range of applications in the agricultural, food, cosmetics and pharmaceutical industries (Naughton et al. 2019; Banat et al. 2010; Nitschke and Costa 2007) due to their surface-active properties (Deleu et al. 1999) and anti-microbial activity (de Araujo et al. 2016; Zhao et al. 2017). Furthermore, surfactin could be established as an emulsifier for food applications (Hoffmann et al. 2021b) and is also commercially available as health supplements due to their potential probiotic effect (Elshaghabee et al. 2017). To elucidate the full potential of process improvement, an in-depth understanding of microorganism-specific control mechanisms is required (Koutinas et al. 2012) as limitations often interfere with optimization of the process (Liu et al. 2017). Although the dependence of the surfactin biosynthesis on the ComQXPA system is well known, studies are often restricted to a qualitative molecular approach and only little quantitative data is available (Dogsa et al. 2021). Therefore, the aim of this study was to establish a profound knowledge of ComX progression and its relation to surfactin productivity during bioreactor cultivations. A reference process of natural surfactin producer *B. subtilis* DSM 10^ T^ was used as a model to exploit the potential of process development beyond the scope of genetic engineering. Batch fermentation processes in pilot scale bioreactor systems were performed with varying glucose concentrations to gather data during different glucose consumption patterns and growth phase lengths. Furthermore, the coherence of peptidases, ComX activity and produced surfactin was examined with special regard on their stability or potential degradation, respectively. After gaining an in-depth knowledge, the information was used to derive a mathematic model for the progression of ComX. By investigating the relation between the ComX pheromone and surfactin production during the fermentation process, a potential for molecular process control strategies can be envisioned.

## Material and Methods

### Chemicals and standards

Chemicals were mainly obtained from Carl Roth GmbH & Co. KG (Karlsruhe, Germany). All chemicals were of analytical grade, if not indicated otherwise. Reference standards for HPTLC measurements for surfactin (≥ 98% purity) and glucose (≥ 99.5% purity) were obtained from Sigma–Aldrich Laborchemikalien GmbH (Seelze, Germany). Azocasein was obtained from Megazyme Ltd. (Wicklow, Ireland). The unmodified ComX peptide backbone (ADPITRQWGD, ≥ 99% purity) was obtained from Thermo Fisher Scientific (Rockford, USA).

### Microorganism and strain maintenance

The microorganisms used in this study are listed in Additional file 1: Table S1. *B. subtilis* wild-type strain DSM 10^ T^ was mainly used for cultivation experiments and strain CT2 for ComX pheromone bioactivity assay. For storage at − 80 °C, cells were cultivated to exponential growth phase and glycerol stocks in lysogeny broth (LB) were prepared containing 15–20% (*v*/*v*) glycerol.

### Construction of mutant strains

Genetic engineering was performed using standard molecular techniques (Harwood and Cutting 1990). Primers (Eurofins Genomics Germany GmbH, Ebersberg, Germany) and plasmids used in this study are listed in Additional file 1: Tables S2 and S3, respectively. For ComX pheromone bioactivity assay, a reporter strain, namely CT2 was engineered. Therefore, a chromosomal integration of P_*srfA*_-*lacZ* fusion was introduced into the *amyE* locus of BKK31700 (Koo et al. 2017), carrying a deletion of *comX* (∆*comX*::*kan*), using the plasmid pKAM446 (Hoffmann et al. 2021a). The mutant strains were selected on LB agar plates supplemented with kanamycin (5 µg/mL), spectinomycin (100 µg/mL) or erythromycin (5 µg/mL), respectively. All plates were incubated at 37 °C. Successful integration disrupted the *amyE* gene, resulting in a loss of amylase activity. This was confirmed with LB agar plates that were supplemented with 1% (*w*/*v*) starch and stained with Lugol's iodine. The genotype verification was performed by PCR (peqSTAR 96X VWR GmbH, Darmstadt, Germany) using Q5® Hot Start High-Fidelity DNA Polymerase (New England Biolabs GmbH, Frankfurt am Main, Germany). Sanger sequencing (Eurofins Genomics Germany GmbH, Ebersberg, Germany) revealed a correct chromosomal integration without any point mutations.

### Media

LB medium was prepared containing 10 g/L tryptone, 10 g/L NaCl and 5 g/L yeast extract. For LB agar plates, LB medium was supplemented with 12.5 g/L bacteriological agar (Bertani 1951). A mineral salt medium (MSM) for enhanced surfactin production was prepared, which is based on the Cooper medium (Cooper et al. 1981) and further improved by Willenbacher et al. (2015). Varying glucose concentrations of 8, 20, 40 and 60 g/L were employed. The buffer concentration was 0.07 M (0.03 M KH_2_PO_4_ and 0.04 M Na_2_HPO_4_) for shake flask cultivation and 0.01 M (4.29 × 10^−3^ M KH_2_PO_4_ and 5.71 × 10^−3^ M Na_2_HPO_4_) for bioreactor cultivation (Willenbacher et al. 2014). The pH of the media was adjusted to pH 7.0 for shake flask cultivation prior to sterilization with the autoclave (15 min, 1 bar, 121 °C). In case of tryptophan auxotrophy, sterile filtrated tryptophan (50 µg/mL) was added to the cultivation medium. For cultivation of the reporter strain CT2, the MSM (8 g/L glucose) was supplemented with spectinomycin (100 µg/mL).

### Preparation of inoculum cultures

For cultivation experiments, incubation was performed at 120 rpm and 37 °C in an incubator shaker (Newbrunswick™/Innova® 44, Eppendorf AG, Hamburg, Germany), unless otherwise stated. For preculture I, 20 mL LB medium were inoculated with 100 µL of the respective glycerol stock in a 100 mL baffled shake flask and incubated for 15–16 h. Preculture II was performed in the respective MSM used for later cultivation experiments and inoculated with preculture I to a starting OD_600_ of 0.1. The filling volumes varied, depending on the cultivation experiment. Typically, 50 mL of MSM was used in a 250 mL baffled shake flask for subsequent shake flask cultivations and 200–300 mL in 2000–3000 mL shake flasks for bioreactor cultivations. Preculture II was incubated for 10–15 h to reach exponential phase before inoculating the main culture. Precultures of strain CT2 for bioassay determinations were prepared with minor adjustments, thus allowing easier handling. The incubation times were extended by decreasing the temperature to 30 °C and by inoculating preculture II to an initial OD_600_ of 0.05. This resulted in incubation times of 24 h for preculture I and 16 h for preculture II.

### Cultivation conditions

Shake flask cultivations were carried out in baffled shake flasks. Main cultures were operated with relative filling volumes of 0.1 mL/mL (10%) in respective MSM and inoculated with preculture II to a starting OD_600_ of 0.1.

Bioreactor cultivations were carried out in 42 L custom-built bioreactors (ZETA GmbH, Graz/Lieboch, Austria) in batch mode with a filling volume of 20 kg. The parameter settings were partially based on a previously published batch process for strain DSM 10^ T^, as described by Willenbacher et al. (2014; 2015). The dissolved oxygen was set to a minimum of 20% and was consistently regulated, with an initial agitation rate of 300 rpm (Rushton turbine) and an aeration rate of 1.4 L/min, corresponding to 0.07 vvm. Temperature was set to 37 °C and pH was kept constant at 7.0 using 4 M NaOH and 4 M H_3_PO_4_. Both a mechanical and a chemical strategy were used to control the intense foaming, as illustrated by Klausmann et al. (2021). A sensor in the headspace of the bioreactor first activated a foam centrifuge set to 2790 rpm. A second sensor in the exhaust line controlled the addition of antifoam agents (Contraspum A4050; Zschimmer & Schwarz GmbH, Lahnstein, Germany and Xiameter® AFE-1520; Dow Silicones Corporation, Midland, USA), which was kept to a minimum (~ 40–60 mL). To prevent blockage of the exhaust filter in the event of potential over-foaming, a 50 L foam trap was connected upstream.

### Sampling and sample analysis

Samples for offline measurements were taken regularly starting from the beginning of the cultivation (*t*_0 _= 0 h) at intervals between 2 and 8 h. The OD_600_ was determined immediately (Biochrom WPA CO8000, Biochrom Ltd., Cambridge, UK) and samples were centrifuged for 10 min at 4816 g and 4 °C (Heraeus X3R, Thermo Fisher Scientific GmbH, Braunschweig, Germany). If necessary, centrifugation was repeated until a clear supernatant was obtained, which was then stored at − 20 °C until further analysis. Cell dry weight (CDW) was calculated from OD_600_ by using a correlation factor of 3.2 ± 0.3 (10.3% RSD), which was determined as described by Geissler et al. (2019a). The cell-free supernatant was analyzed for surfactin, glucose, and ammonium concentrations, as well as peptidase and ComX activity. Thereby, the glucose and surfactin concentrations were determined using a High-Performance Thin-Layer Chromatography (HPTLC) system (CAMAG Chemie-Erzeugnisse und Adsorptionstechnik AG, Muttenz, Switzerland) as described by Geissler et al. (2017, 2019a). For surfactin detection, 1 mL of sampled cell-free supernatant was extracted thrice with 1 mL of chloroform/methanol (2:1; *v*/*v*). After evaporation for 40 min at 10 mbar and 40 °C (RVC2-25 Cdplus, Martin Christ Gefriertrocknungsanlagen GmbH, Osterode am Harz, Germany), the sample was resuspended in 1 mL methanol and applied on HPTLC Silica gel 60 plates (Merck KGaA, Darmstadt, Germany). Development was conducted using a mobile phase of chloroform/methanol/water (65:25:4; *v*/*v*/*v*) and surfactin was detected at 195 nm. For glucose detection, no extraction step was required, and the cell-free supernatant could be directly applied on HPTLC Silica gel 60 F254 plates (Merck KGaA, Darmstadt, Germany). Development was conducted using acetonitrile/water (85:15; *v*/*v*) as mobile phase. After a derivatization step with diphenylalanine (DPA reagent), glucose was detected at 620 nm. Quantification of ammonium was performed photometrically using the Spectroquant® Ammonium Assay Kit (Cat. No.: 114752, Merck KGaA, Darmstadt, Germany) according to the manufacturer's instructions in 96-well plates with a reduced volume of 5%. Calibration range was defined from 0.05 to 4.0 mg/L for the reduced volume.

### ComX pheromone bioactivity assay (ComX bioassay)

Due to the high complexity of ComX purification, the pheromone was measured with a bioassay based on the principle provided by Magnuson et al. (1994). Thereby, reporter strain CT2 (∆*comX*::*kan*; P_*srfA*_-*lacZ*) can be used as an assay reagent and a ComX-dependent expression of *lacZ* can be determined by measuring the β-galactosidase activity with the Miller assay (Miller 1972). The employed bioassay is further in line with a recently published method, provided by Dogsa et al. (2021). A detailed description of the working principle and further validation experiments are given in the supplementary information. In brief, a main culture of strain CT2 was inoculated with preculture II to a starting OD_600_ of 0.1 and incubated for 3 h at 120 rpm and 37 °C. 750 µL of the pre-incubated main culture were then mixed with an equal volume of sampled cell-free supernatant and incubated in culture tubes for 3 h (shake flask cultivation) and 5 h (bioreactor cultivation) at 120 rpm and 37 °C. In case of an expected high peptidase activity in the samples, BSA (50 µg/mL) was added to prevent ComX from potential degradation due to extracellular proteases (Magnuson et al. 1994; Spacapan et al. 2018). After measuring the OD_600_, the samples were used for β-galactosidase assay with means of the Miller assay, according to the protocol described by Hoffmann et al. (2020). The ComX activity is expressedn Miller units (MU) and represents the highest possible induction of P_*srfA*_ as a function of the present ComX concentration (Eq. 1).

1 $${\text{MU}} = 1000 \cdot \frac{{{\text{OD}}_{{420{\text{nm}}}} - \left( {1.75 \cdot {\text{OD}}_{{550{\text{nm}}}} } \right)}}{{{\text{t}} \cdot {\text{v}} \cdot {\text{OD}}_{{600{\text{nm}}}} }}$$

where *t* represents the incubation time and *v* the sample volume. Bnk values were measured using the respective sterile cultivation medium instead of sampled cell-free supernatant. The limit of detection (LOD) and limit of quantification (LOQ) was calculated from 20 samples using Eqs. 2 and 3 (Shrivastava and Gupta 2011).

2 $${\text{LOD}} = {\overline{\text{m}}}_{{\text{B }}} + 3{\text{S}}_{{\text{B}}} .$$

3 $${\text{LOQ}} = {\overline{\text{m}}}_{{\text{B }}} {\text{ + 10S}}_{{\text{B}}}$$

where ${\stackrel{\mathrm{-}}{\text{m}}}_{\text{B}}$ represents the mean value of the blank and ${\text{S}}_{\text{B}}$ the respective standard deviation. For selection of blank values, a Shapiro–Wilk test was performed, which confirmed a normal distribution, with p ≥ 0.559 for shake flask cultivations and p ≥ 0.200 for bioreactor cultivations. Based on this, values with a z-score ≥ 2.0 were excluded from the calculation. This resulted in an LOD of 23.9 MU and LOQ of 42.7 MU for shake flask cultivations and a LOD of 30.3 MU and a LOQ of 55.4 MU for bioreactor cultivations.

### Liquid chromatography-mass spectrometry analysis of ComX pheromone

Cell-free supernatant was adjusted to pH 2 by adding 6 N HCl and samples were incubated for 1 h at 4 °C to precipitate the ComX pheromone. Samples were then centrifuged for 10 min at 21,000 g at room temperature (Centrifuge 5415 D, Eppendorf AG, Hamburg, Germany). The supernatant was discarded, and the pellet was dissolved in 100% methanol. The methanolic solution was centrifuged for 10 min at 21,000 g at room temperature and the supernatant was used for liquid chromatography-mass spectrometry (LC–MS) analysis. Therefore, a 1290 UHPLC system (Agilent Technologies, Waldbronn, Germany) coupled to a Q-Exactive Plus Orbitrap mass spectrometer equipped with a heated electrospray ionization (HESI) source (Thermo Fisher Scientific GmbH, Bremen, Germany) was used. Chromatographic separation of methanolic extracts from *B. subtilis* strains was performed on a CSH C18 150 mm × 1 mm column (1.7 μm particle size, Waters GmbH, Eschborn, Germany). The column temperature was maintained at 55 °C. In total, 10 µl of each methanolic extract was injected. A synthetic unmodified ComX peptide was used for building a calibration curve. For mobile phase A, 0.1% formic acid in water, and for mobile phase B 0.1% formic acid in acetonitrile was used. A constant flow rate of 0.12 ml/min was used and the gradient elution was performed as follows: 20–35% B from 0 to 3 min, –65% B from 3 to 4 min, 35–100% B from 4 to 13 min, 100% B from 13 to 16 min. The system was returned to initial conditions from 100 to 20% B from 16 to 17 min. The Q-Exactive Plus mass spectrometer was calibrated externally using the manufacturer’s calibration solutions (Pierce™, Thermo Fisher Scientific GmbH, Bremen, Germany). The HESI source was operated in positive ion mode, with a spray voltage of 4.2 kV and an ion transfer capillary temperature of 290 °C. Sweep gas and auxiliary pressure rates were set to 15 and 2, respectively. The S-Lens RF level was 50% and the auxiliary gas heater temperature was 200 °C. A targeted single ion monitoring method including data dependent MS2 scans (tSIM/ddMS2) was used for quantification of the ComX pheromone. The *m*/*z* ratios of the + 2 charged precursor ions of the unmodified ComX peptide ([M + 2H]^++^, *m*/*z* 579.78050) and farnesylated ComX peptide (M + 2H]^++^, *m*/*z* 681.87470) were used as predefined target ions. Mass spectra were acquired in SIM mode with an isolation window of 1.6 Da at a resolution of 70,000 FWHM, and an Automatic Gain Control (AGC) target of 3 × 10^6^ and 100 ms maximum ion injection time (MIT). Data dependent MS2 spectra of precursor ions were acquired with a resolution of 17,500 FWHM, an AGC target of 5 × 10^5^, 100 ms MIT and a normalized collision energy of 30. An exemplary extracted ion chromatogram (XIC) and an ESI–MS/MS spectrum can be found in the supplementary material (Additional file 1: Figure S1).

### Endopeptidase activity

The activity of extracellular proteases was determined by measuring the endopeptidase activity with the azocasein assay. The method was adapted from Baur et al. (2015), following a measurement principle based on Charney and Tomarelli (1947). To reflect the activity of the peptidases during cultivation as realistically as possible, the parameters were approximated to the cultivation conditions. For this purpose, 5 g/L sulfanilamide azocasein solution was dissolved in MSM buffer of the analyzed cultivation and adjusted to pH 7.0. 100 µL pre-incubated substrate solution (5 min at 40 °C) was added to 100 µL of cell-free supernatant. The mixture was incubated for 1 h at 800 rpm and 37 °C and the reaction was stopped with 20 µL of 2 M TCA. After centrifugation for 10 min at 19,357 g and 4 °C (Centrifuge 5430R, Eppendorf AG, Hamburg, Germany), 150 µL of the solution were transferred to a 96-well microtiter plate. Before the absorption was measured at 450 nm, 50 µL of 1 M NaOH were added. It was verified by means of time-turnover curves (data not shown) that measurements were within linear range and samples were diluted accordingly. For blank measurements, the cell-free supernatant was added after reaction stop using TCA. The volumetric endopeptidase activity [EA_peptidase_] is defined as the absorbance difference [∆A] between sample and blank at 450 nm per h and mL cell-free supernatant [∆A/(h·mL)].

### Degradation experiments

Cell-free supernatant was obtained from bioreactor cultivation with 40 g/L glucose by two centrifugation steps for 30 min at 4816 g and 4 °C (Heraeus X3R, Thermo Fisher Scientific GmbH, Braunschweig, Germany). The time points were selected to represent the exponential phase, the phase around the highest ComX activity, and the stationary phase, around CDW_max_, resulting in different peptidase activities at each time point. The supernatant was then filtered using a filter with a retention capacity of 7–12 µm. To prevent further growth, the supernatant was treated with spectinomycin (100 µg/mL), as sterile filtration was not possible, due to immediate filter blockage. Cell-free supernatant was incubated in baffled shake flasks with a relative filling volume of 10%. Incubation was performed in biological duplicates at 120 rpm and 37 °C. Samples were taken after *t* = 0, 1, 2, 3, 4, 6 and 8 h and analyzed for ComX and peptidase activity and the ComX degradation rate in (MU/h) was calculated from the measured data.

For autodegradation studies, strain DSM 10^ T^ was additionally cultivated in 3000 mL shake flasks using MSM with 40 g/L glucose. The supernatant was collected after 14 h of cultivation, corresponding roughly to the highest peptidase activity in DSM 10^ T^. The supernatant was prepared as previously described and additionally sterile filtered (2 µm) to prevent growth and incubated for 30 min at 80 °C to inactivate proteases. To mimic bioreactor conditions, the pH was adjusted to 7.0. The incubation was performed as previously described in a biological triplicate. To gather information about the influence of the cell fraction on putative ComX degradation, a ComX deficient strain was cultivated in spent medium. Therefore, *B. subtilis* BKK31700 (∆*comX*) and the corresponding wild-type strain *B. subtilis* DSM 23,778 were both cultivated in MSM, employing 8 g/L glucose. The cells were harvested after 12.5 h by two-fold centrifugation for 15 min at 4816 g and 4 °C (Heraeus X3R, Thermo Fisher Scientific GmbH, Braunschweig, Germany). The cells of strain BKK31700 were washed in between the centrifugation process with sterile saline solution (0.9% *w*/*v*) and resuspended in an equal volume of spent filtered medium of strain DSM 23,778. To prolong cultivation time, 4 g/L glucose were added to the culture. The incubation was performed in biological duplicates for 24 h at 120 rpm and 37 °C and samples were taken regularly and analyzed for OD_600_, ComX and peptidase activity.

### Data analysis and process parameters

For evaluation of the cultivations, the biomass yield per substrate *Y*_X/S_ [g/g], the product yield per substrate *Y*_P/S_ [g/g], the product yield per biomass *Y*_P/X_ [g/g] as well as the specific growth rate *µ* [1/h] and the specific productivity *q* [g/(g·h)] were determined using Eqs. 4, 5, 6, 7, and 8 (Geissler et al. 2019a). Process parameters were calculated for the respective replicates using absolute values at the time point when at least 90% of the maximum value was exceeded. Hence, *Y*_X/S_ was calculated at X_≥90%_, *Y*_P/S_ at P_≥90%_ and *Y*_P/X_ and *q*_overall_ at P_≥90%_ and X_≥90%_ with *t*_0_ used as first time point.

4 $$Y_{{{\text{X}}/{\text{S}}}} = \frac{{\Delta m_{{\text{X}}} }}{{\Delta m_{{\text{S}}} }}$$

5 $$Y_{{{\text{P}}/{\text{S}}}} = \frac{{\Delta m_{{\text{P}}} }}{{\Delta m_{{\text{S}}} }}$$

6 $$Y_{{{\text{P}}/{\text{X}}}} = \frac{{\Delta m_{{\text{P}}} }}{{\left( {\frac{{\left( {m_{{{\text{X}}_{1} }} + m_{{{\text{X}}_{2} }} } \right)}}{2}} \right)}}$$

where X represents the biomass, P the product, here surfactin, and S e substrate, here glucose.

7 $$\mu = \frac{{\ln \left( {\frac{{m_{{{\text{X}}2}} }}{{m_{{{\text{X}}1}} }}} \right)}}{{\left( {t_{2} - t_{1} } \right)}}$$

Thereby *µ*_max_ is defined as the maximum growth rate.

8 $$q = \frac{{\Delta m_{{\text{P}}} }}{{\left( {\frac{{\left( {m_{{{\text{X}}_{1} }} + m_{{{\text{X}}_{2} }} } \right)}}{2}} \right) \cdot \Delta t}}$$

The overall specific productivity *q*_overall_ determined the entire process, while *q*_max_ is defined as the maximum specific productivity.

### Modeling platform and software

All cultivation experiments were carried out in biological duplicates or triplicates. For bioreactor cultivations, the offline measurements were additionally carried out in technical duplicates, yielding in at least 4 measurements for each point in time. Mathematic modeling was performed using programming and numeric computing platform MATLAB (The MathWorks Inc., Natick, USA). As described previously (Henkel et al. 2013), all models were implemented as nonlinear differential equations. For simulation of the ordinary differential equations the ODE-solvers “ode23s” and “ode15s” embedded in the MATLAB environment were used. Nonlinear parameter optimizations and fitting were performed using the “fmincon” functions. A least-square error function was assumed for all parameter optimizations. Graphs were generated from obtained results using the scientific graphing and data analysis software Sigma Plot (Systat Software Inc., San Jose, USA), which was also used for statistical analysis. The experimentally recorded carbon and nitrogen data were fitted using a sigmoidal or logistic curve fit with 4 parameters. To correlate specific surfactin productivity *q*_surfactin_ to ComX, the experimentally plotted data for biomass, surfactin and ComX activity were fitted using either a sigmoidal curve fit with 4 parameters or an exponential fit with 3 parameters. In this case, the data range was limited up to ComX_max_ and specific productivity *q*_surfactin_ could be calculated from the fitted values. Xcalibur™ software version 4.4.16.14 (Thermo Fisher Scientific, San Jose, USA) was used for data acquisition and data analysis of LC–MS measurements.

## Results

### Time course of ComX activity during a surfactin reference process

To take a closer look at ComX-mediated surfactin production during a bioprocess, cultivations were performed in a pilot scale bioreactor system. A reference process with natural surfactin producer *B. subtilis* DSM 10^T^ was cultivated in optimized MSM (Willenbacher et al. 2015), employing 40 g/L glucose to cover a full growth curve (Fig. 2). After 32 h of cultivation, the highest CDW of 5.4 ± 0.4 g/L was achieved, resulting in a *Y*_X/S_ of 0.15 g/g and *µ*_max_ of 0.52 1/h (Table 1). After reaching glucose depletion at approximately 40 h, a decline phase was observed. Throughout the whole process, ComX activity and surfactin concentration were determined and are shown in the middle and lower part of Fig. 2. The highest surfactin titer was reached after 40 h with P_max_ = 1346.6 ± 100.3 mg/L, resulting in an *Y*_P/X_ of 0.49 g/g and an overall specific productivity of *q*_overall_ = 0.02 g/(g·h). An increase in biological ComX activity was observed during exponential growth phase, peaking towards the end of this phase. The high-point in the ComX time course was reached when cell growth first stagnated, with 215.9 ± 17.6 MU after 16 h of cultivation. Thereafter, the ComX activity declined and showed a tendency to oscillate, settling at a value of approximately 150–160 MU.

**Table 1:** Overview of process parameters of bioreactor batch cultivations, including yield coefficients, highest surfactin titer P_max_ [mg/L], highest biomass X_max_ [g/L], highest ComX activity ComX_max_ [MU], as well as the ComX activity at the end of the cultivation ComX *t*_end_ [MU]

Cultivation	Bioreactor cultivation					
Parameter	20 g/L		40 g/L		60 g/L	
X_max_ [g/L]	5.7 ± 0.6	20 h	5.4 ± 0.4	32 h	5.0 ± 0.1	24 h
P_max_ [mg/L]	800.5 ± 81.3	48 h	1346.6 ± 100.3	40 h	885.7 ± 224.2	44 h
ComX_max_ [MU]	205.6 ± 4.0	20 h	215.9 ± 17.6	16 h	168.3 ± 11.6	12 h
ComX [MU] *t*_end_	163.7 ± 20.5	48 h	134.2 ± 19.9	44 h	131.3 ± 6.6	48 h
*Y*_X/S_ [g/g] at X_≥90%_	0.29		0.15		0.21	
*Y*_P/S_ [g/g] at P_≥90%_	0.04		0.04		0.03	
*Y*_P/X_ [g/g] at P_≥90%_/X_≥90%_	0.26		0.49		0.33	
*µ*_max_ [1/h]	0.71		0.52		0.66	
*q*_overall_ [g/(g·h)]	0.01		0.02		0.01	

### Correlation of specific productivity and ComX activity in 40 g/L batch cultivation

To establish a correlation between the measured ComX activity by bioassay and the present ComX concentration, selected samples of the approach with 40 g/L glucose (replicate 1) were analyzed by LC–MS. A comparable time course of the ComX activity and the determined ComX concentration was identified and is illustrated in Additional file 1: Figure S2a. The sample with the highest measured ComX concentration of 4.5 µg/L resulted in a ComX activity of 230.5 ± 2.7 MU with respect to the bioassay. This is equivalent to a molar concentration of 3.3 nM and was achieved after 14 h of cultivation at an CDW of 2.7 ± 0.1 g/L. The specific surfactin productivity *q*_surfactin_ initially increased with increasing ComX activity until *q*_max_ = 0.27 g/(g·h) was reached (Fig. 3a). Subsequently, *q*_surfactin_ declined despite further rising ComX activity. In fact, *q*_max_ was already reached between 8 and 9 h of cultivation at a ComX activity of 136.5 MU.

### Degradation of ComX pheromone

**Fig. 1:**
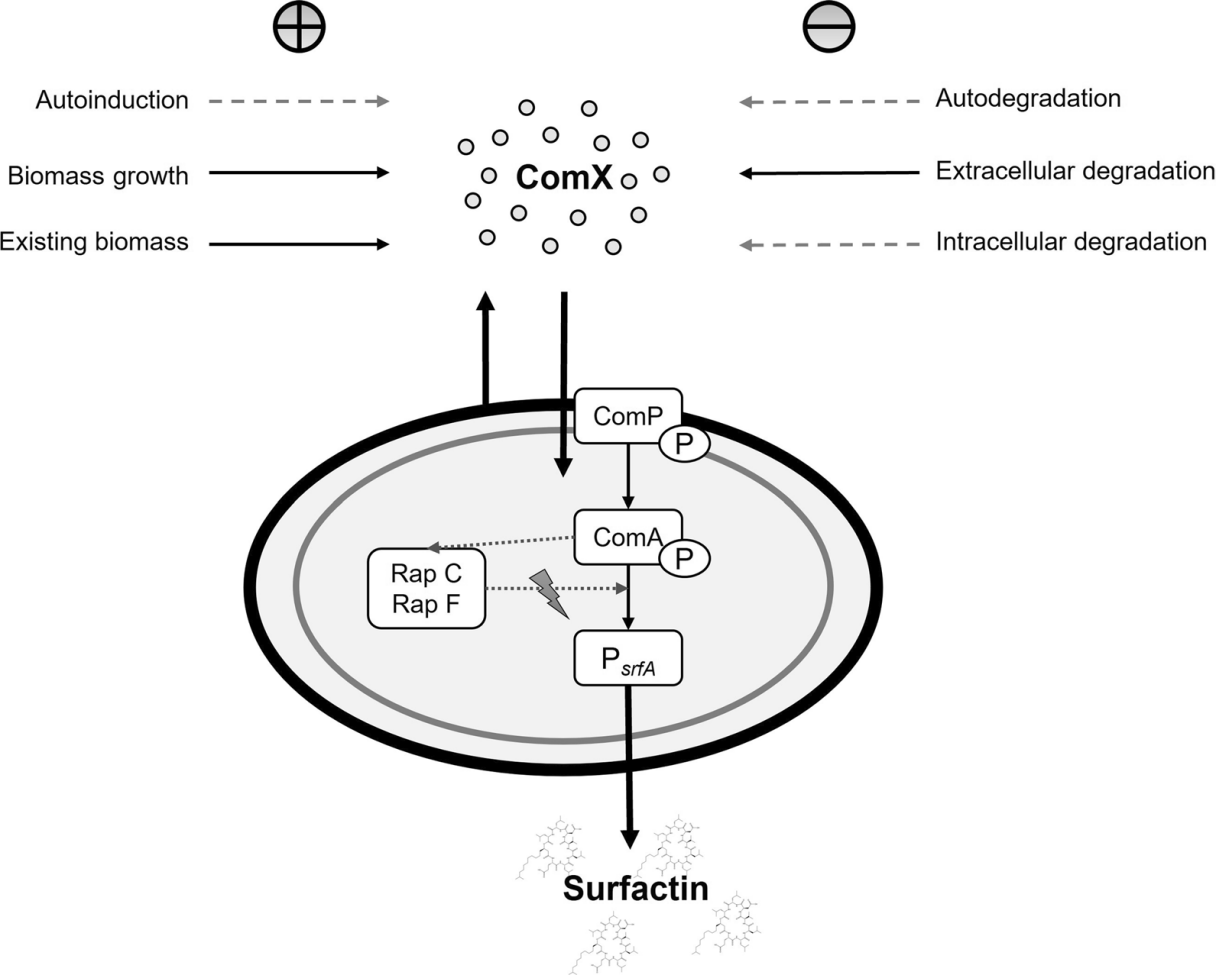
Schematic illustration of potential influencing factors on ComX concentration and surfactin production**.** The terms included in the here presented model for ComX time course are marked with a solid line

**Fig. 2:**
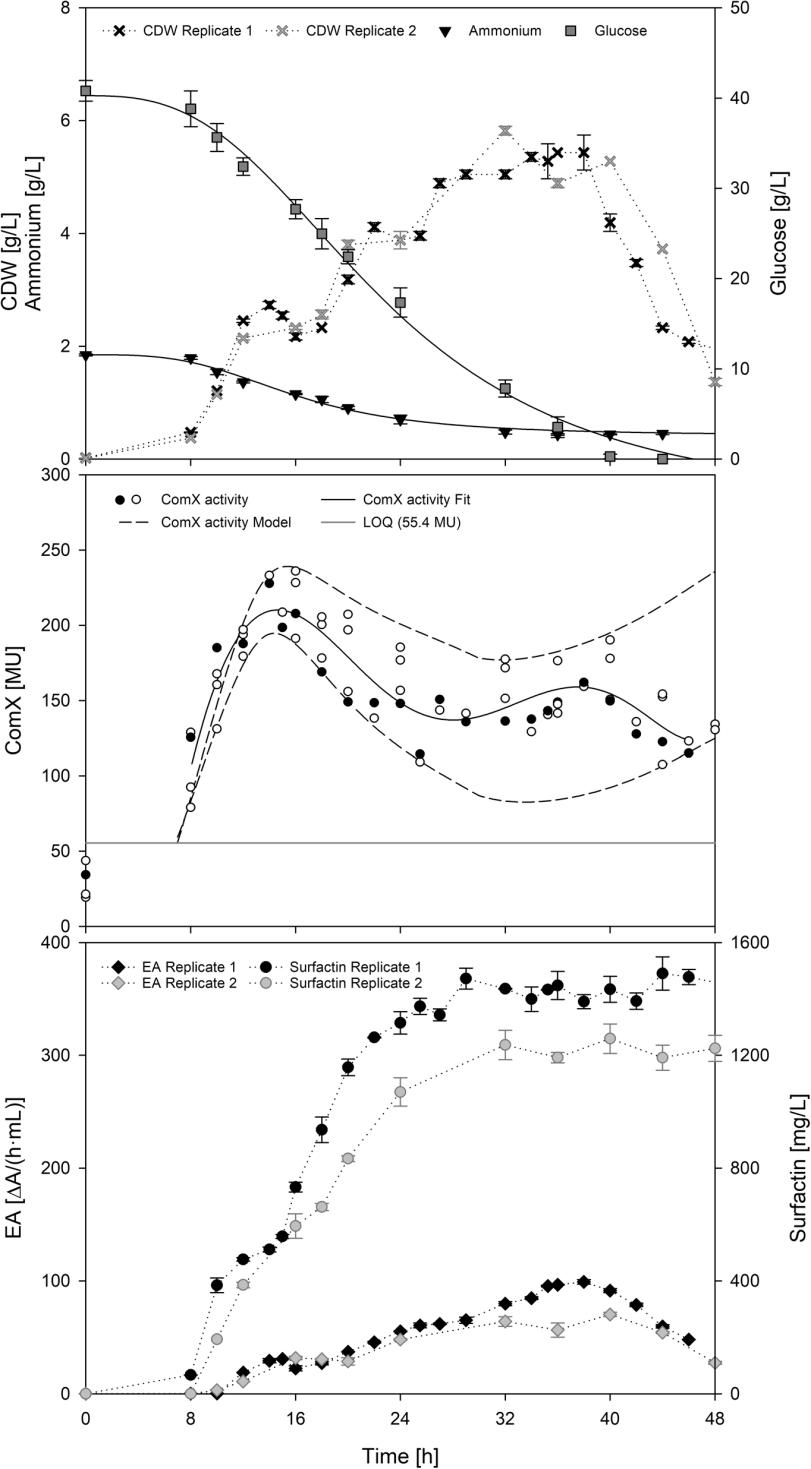
Time course of bioreactor batch cultivation of *B. subtilis* DSM 10^ T^ employing 40 g/L glucose. ComX activity (white circle) is shown in the middle of the graph. The model was used to successfully describe the trajectory of one set of experimental data (black line). A high sensitivity to changes in parameters is demonstrated, as variation of parameter value less than 8% allowed for description of the entire cloud of all available data from biological and technical replicates using parameter values from Table 2 (dashed lines) from the beginning of maximum ComX activity

**Fig. 3:**
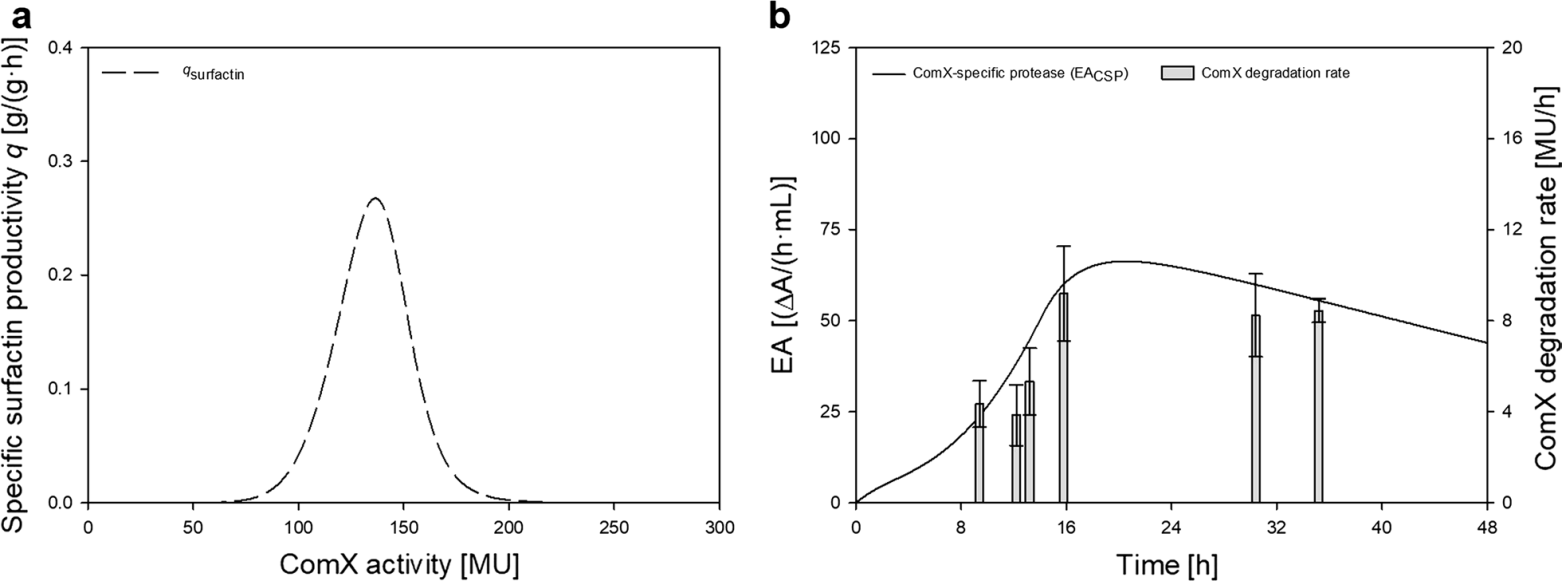
**a** Specific surfactin productivity *q*_*Surfactin*_ (dashed line) in relation to ComX activity; **b** Time course of modeled peptidase activity of putative ComX-specific protease EA_CSP_ (black line) and ComX degradation rates (gray bar) plotted against the cultivation time

The LC–MS analysis of extracellular ComX also confirmed degradation after reaching a peak during cultivation process, which was also detected by the bioassay (Additional file 1: Figure S2a). Resembling Henkel et al. (2013), experiments were performed with respect to (*i*) autodegradation, (*ii*) extracellular degradation and (*iii*) intracellular degradation (Fig. 1), to obtain deeper understanding of the degradation kinetics of ComX. As reported by Spacapan et al. (2018) and Magnuson et al. (1994), ComX appears sensitive to extracellular proteases, especially serine proteases. Therefore, endopeptidase activity was determined over the course of cultivation (Fig. 2, bottom). To determine possible degradation as a function of peptidase activity (Fig. 3b), the supernatant was incubated at different time points throughout the cultivation with special emphasis on the exponential phase, the phase around ComX_max_ and the phase around CDW_max_. Interestingly, the total endopeptidase activity (EA_total_) correlated with cell density and not with determined degradation rates (Fig. 3b), as these stagnated despite a further increase in total endopeptidase activity (Fig. 2). The highest EA_total_ = 80.9 ± 10.7 ∆A/(h·mL) was determined after 40 h and was thus approximately in the time frame of CDW_max_. After reaching the highest cell density, a simultaneous decline of CDW and EA_total_ was observed. In comparison, the highest ComX degradation rate of 9.2 ± 2.1 MU/h was determined after approximately 16 h of cultivation, which corresponded to the time of highest ComX activity. As cultivation continued, degradation rates stagnated and even showed a downward trend, reaching 8.4 ± 0.5 MU/h after approximately 35 h of cultivation. Spacapan et al. (2018) hypothesized that ComX-induced extracellular proteases are responsible for ComX degradation. To test this hypothesis, the following model for a putative ComX-specific protease (CSP) was established (Eq. 9). The according parameters are listed in Table 2.

9 $$\frac{{{\text{d}}EA_{{{\text{CSP}}}} }}{{{\text{d}}t}} = f \cdot a_{{{\text{ComX}}}} - g \cdot EA_{{{\text{CSP}}}}$$

**Table 2:** List of parameter values, units and description used for modeling

Parameter	Value	Unit	Mode	Function
*a*_ComX_ at *t*_0_	21–32	MU	Range	Initial ComX activity
*b*	17.72–19.95	(MU·L)/(g·h)	Range	Biomass-dependent ComX formation
*d*	0.436–0.516∙10^3^	(MU·L)/g	Range	Growth-dependent ComX formation
*e*	1.36–1.58	MU/(EA·h)	Range	CSP-dependent ComX degradation
*f*	0.115	EA/(MU·h)	Fixed	CSP formation
*g*	0.328	1/h	Fixed	CSP degradation

The modeled enzyme activity of CSP (EA_CSP_) is presented in Fig. 3b. Here, an increase in EA_CSP_ was initially observed and, after reaching a maximum, a slight downward trend was seen, which was also accompanied by calculated degradation rates. The highest modeled EA_CSP_ = 66.3 ∆A/(h·mL) was reached after 20.5 h, which is concomitant to the highest measured degradation rate after 16 h. As shown in Fig. 2, the most pronounced degradation of ComX activity was also observed in this time frame, after the peak of ComX activity was reached after 16 h. However, to unveil a potential autodegradation effect, a heat-treatment for 30 min at 80 °C of the supernatant was performed to reduce the influence of extracellular proteases. Thereafter, the remaining peptidase activity was at an average value of 0.2 ± 0.0 ∆A/(h·mL) (Additional file 1: Figure S2b), compared to the control group with an average of 52.1 ± 2.1 ∆A/(h·mL) (data not shown). The treated supernatant was incubated in baffled shake flasks and assayed for ComX activity (Additional file 1: Figure S2b). After 8 h of incubation, a remaining ComX activity of 251.1 ± 17.1 MU was determined. Compared to the initial ComX activity at *t*_0_ of 268.6 ± 12.7 MU, this exhibited an activity loss of 6.5%. Dogsa et al. (2021) further only observed a degradation effect of purified ComX after 100 days of storage. Similarly, Magnuson et al. (1994) stated a heat stability of ComX for 12 h at 80 °C. Taken this and the here demonstrated activity loss of less than 10% together, it can be assumed that ComX autodegradation is relatively low. In addition to that, a potential intracellular degradation caused by the cellular fraction was investigated. As no commercial standard ComX peptide was available, the experiment was performed by culturing a ComX deficient strain in spent medium containing ComX from the same *Bacillus* subsp. (Additional file 1: Figure S2c). A potential degradation caused by the cells should have been detected as the ComX deficient strain is not able to produce its own ComX. However, after 24 h of cultivation a ComX activity of 103.2 ± 11.4 MU remained, which was comparable to an initial ComX activity of 96.5 ± 3.0 MU at *t*_0_. These results gave reason to assume that intracellular degradation was also at a low level.

### A model for the time course of ComX

As illustrated in Fig. 1, different factors can play a role on the ComX time course. Thereby, the ComX production might be influenced by (*i*) autoinduction, (*ii*) biomass growth and (*iii*) existing biomass. The reliance of P_*srfA*_ activity on the cell density has recently been confirmed (Dogsa et al. 2021). An auto inductive term can however presumably be excluded because, to the best of our knowledge, no regulatory mechanism for the ComQXPA system is known so far. This assumption is also supported by literature findings, as Bacon Schneider et al. (2002) hypothesized the absence of ComX autoinduction and Dogsa et al. (2021) proposed a signal-response model for ComX production as a square function of the biomass without a positive feedback loop. However, this was not adapted for our process, due to the differently chosen research focus and the restriction to signal-response during early growth stages. Also, as already established, degradation kinetics must be included. However, our own experiments gave no indication that either autodegradation or intracellular degradation occred. For a surfactin reference process we therefore propose a refined model for ComX production (Eq. 10), including the biomass growth and the existing biomass as well as a degradation term based on a putative ComX-specific protease EA_CSP_ (for a detailed description on degradation refer to previous section).

10 $$\frac{{{\text{d}}a_{{{\text{ComX}}}} }}{{{\text{d}}t}} = b \cdot c_{{{\text{CDW}} }} + d \cdot \frac{{ {\text{d}}c_{{{\text{CDW}}}} }}{{{\text{d}}t}} - e \cdot EA_{{{\text{CSP}}}} .$$

The respective parameters and ranges are listed in Table 2. The modeled time course of ComX was subject to a high sensitivity to changes in parameters, as minor adjustments to parameter values less than 8% resulted in extending the model range to include the entire cloud of all available data from all technical and biological replicates (Fig. 2). Furthermore, the model was successfully used to describe the trajectory of one set of experimental data (black line).

### Validation and transfer of the ComX model on bioreactor batch cultivations with varying glucose availabilities

**Fig. 4:**
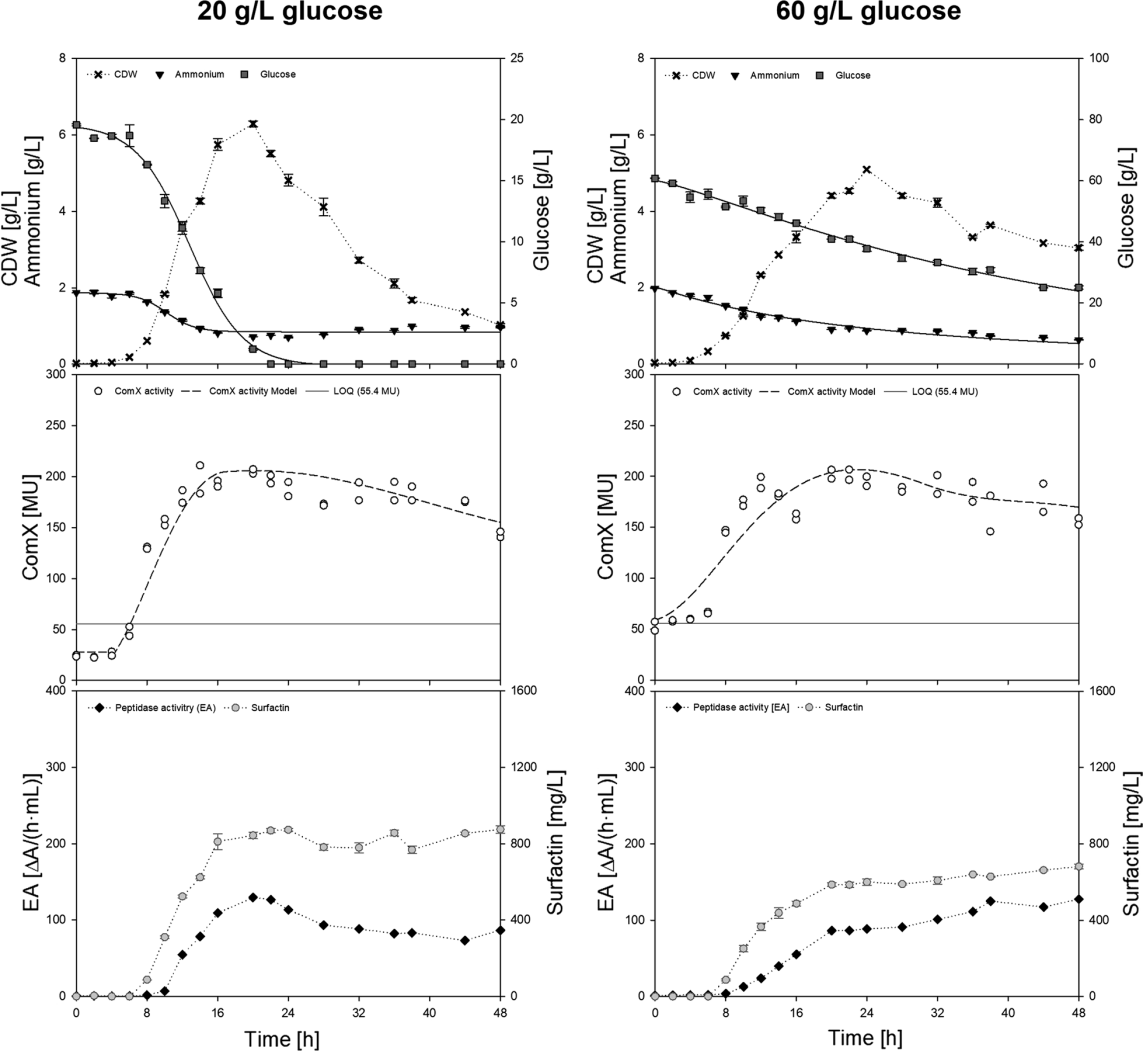
Graphical illustration of exemplary bioreactor batch cultivations of *B. subtilis* DSM 10^ T^ employing 20 and 60 g/L glucose. Each plot represents one of two biological replicates and the ComX activity (white circle) is additionally shown as technical replicate. The dashed black line represents the modeled ComX activity

Bioreactor cultivations with varying glucose concentrations were performed, to transfer the model for ComX to other process scenarios (Fig. 4). Therefore, 20 and 60 g/L glucose were chosen to reflect ± 50% of the initial reference process and to achieve different glucose depletion patterns. Here, a depletion of glucose was reached when using 20 g/L, whereas 60 g/L glucose was not completely consumed. Using 20 g/L of glucose, cells reached a maximum CDW of 5.7 ± 0.6 g/L after 20 h, followed by a decline phase due to glucose depletion. This approach achieved the highest *Y*_X/S_ of 0.29 g/g and the highest *µ*_max_ of 0.71 1/h of all cultivations. In case of the cultivation with 60 g/L, glucose was not completely consumed, with still 21.6 ± 3.5 g/L remaining after 48 h of cultivation. Instead, ammonium was at a low level, which resulted in a prolonged stationary phase and only minor decrease in the CDW after reaching its peak of 5.0 ± 0.1 g/L after 24 h of cultivation. A typical decline phase was not observed in this experimental set-up. The *Y*_X/S_ reached a value of 0.21 g/g and a *µ*_max_ of 0.66 1/h. For both experimental set-ups, the average surfactin titer was in a comparable range with P_max_ = 800.5 ± 81.3 mg/L for the approach with 20 g/L and P_max_ = 885.7 ± 224.2 mg/L for the cultivation with 60 g/L. The ComX time course showed a slightly different trend, compared to the cultivation with 40 g/L of glucose. Again during exponential phase an increase in ComX activity was determined until a maximum of 205.6 ± 4.0 MU was reached for the approach with 20 g/L glucose and 168.3 ± 11.6 MU when emplyoing 60 g/L glucose. However, the decrease of ComX and also the tendency to oscillate was not as pronounced compared to the cultivation with 40 g/L glucose.

## Discussion

As an important step in understanding the behavior of the time course of ComX concentration in *B. subtilis*, Dogsa et al. (2021) recently established a signal-response model. However, the model was restricted to an early growth stage and to the expression of P_*srfA*_, thus again comprising only the molecular level. In the current study, we aimed at extending the model for the ComX time course to the whole surfactin production process in a bioreactor system. The most important findings of this study can be summarized as:

- Novel quantitative insights into the time course of ComX concentration are provided
- ComX activity was peaking towards the transition to stationary phase; highest detected ComX concentration was 3.3 nM
- Specific surfactin productivity *q*_surfactin_ did not increase linearly with ComX activity
- Highest degradation rate (9.2 ± 2.1 MU/h) was found in transition to stationary phase
- Data suggest that autodegradation and intracellular degradation have only a minor influence
- A model for the time course of ComX suggests a putative ComX-specific protease (CSP), which did not correlate directly with overall endopeptidase activity
- The model potentially enables future molecular process control strategies in wild-type applications

To allow for this optimization strategy, profound quantitative knowledge must be gathered as an initial step. This was done in the presented study regarding surfactin production and ComX pheromone progression, providing new insights into this crosstalk. Several bioreactor batch fermentations with different initial glucose concentrations were performed to illustrate different lengths of growth phases of *B. subtilis* DSM 10^ T^. Thereby the general progression of ComX activity was consistent for all bioreactor set-ups, regardless of varying experimental set-ups. During exponential growth, an initial increase in ComX activity was determined, peaking towards the transition to stationary phase. Since surfactin is mainly expressed in the transition phase (Ongena and Jacques 2008), this coherence was expected. The highest ComX concentration measured in bioreactor cultivations was 3.3 nM. This is within the range of published literature values, as a half maximum response is usually achieved at a ComX concentration of 3–4 nM and a saturation at around 10 nM (Magnuson et al. 1994; Okada et al. 2005, 2007; Dogsa et al. 2021). In terms of the interrelation between ComX activity and surfactin production, one would intuitively assume that a high signal of ComX results in a high surfactin concentration. In general, this was found to be true, as the highest overall ComX activity obtained (215.9 ± 17.6 MU) also correlated to the highest overall surfactin concentration (1346.6 ± 100.3 g/L) for the bioreactor cultivation with 40 g/L glucose. In other words, a higher amplitude of ComX during transition phase seemed to be beneficial for the overall surfactin titer when comparing different bioreactor experiments. However, a non-linear relation between *q*_surfactin_ and ComX activity was observed (Fig. 3a), as *q*_max_ was already determined for a ComX activity of 136.5 MU. The highest possible induction of surfactin biosynthesis genes (= highest ComX concentration) does not automatically have to correlate with the highest surfactin biosynthesis capacity. Amongst ComX, various other factors can influence surfactin production, such as nutrient availability (Geissler et al. 2019b), cell differentiation (Hamoen et al. 2003) or pleiotropic regulators such as CodY or transition state regulator AbrB (Jacques 2011). Further consideration must be paid on the transcriptional activator ComA (Fig. 1). In its phosphorylated state, ComA ~ P not only activates surfactin promoter P_*srfA*_ but affects not less than 89 genes, among them *rapC* and *rapF* (Comella and Grossman 2005). These genes encode for response regulator aspartate (Rap) phosphatases RapC and RapF, which in turn suppress the activity ComA ~ P and thus expression of P_*srfA*_ (Jacques 2011; Comella and Grossman 2005). Since ComA is activated in the presence of ComX, a high ComX concentration might be accompanied by an increased Rap phosphatase activity, possibly resulting in a negative feedback mechanism and finally lower surfactin titers and thus lower productivities. These counteractions would mark an interesting approach for future strain improvement using genetic engineering tools. For microbial wild-type applications specifically these findings implement the need to narrow down the range in which ComX is beneficial and thus to find a mechanism on how to control ComX levels. A suitable tool for this is a mathematic description of the biological system, as provided in this study for the time course of ComX. Differences in the ComX signal were mainly observed during late exponential and transition to stationary phase. Concomitantly, a higher peak intensity of ComX activity during late exponential phase was followed by a more prominent decrease phase, which was particularly evident for the bioreactor cultivation with 40 g/L glucose. What was also apparent for all bioreactor cultivations was that, regardless of the maximum value, a tendency for ComX activity to oscillate towards the end of the cultivation was indicated. An oscillatory behavior of quorum sensing signal molecules was already predicted by Kamino et al. (2011) and could indicate the presence of a negative feedback loop and a direct connection to ComX degrading mechanisms. But also, yet unknown regulatory coherences could have an impact and might explain the settling of the ComX activity. Based on the given literature (Magnuson et al. 1994; Spacapan et al. 2018), it can be assumed that proteases might in part be responsible for the fluctuations and degradation of ComX. Spacapan et al. (2018) suggested the possibility of a negative feedback loop by extracellular proteases, specifically those that are induced by ComX itself. Interestingly, stabilization of ComX activity occurred toward the end of each cultivation although overall peptidase activity was still increasing. Preliminary shake flask cultivations drew a similar picture (Additional file 1: Figure S3). In the shake flask cultivation using 8 g/L of glucose, peptidase activity remained consistent and so did the ComX activity. In contrast, when employing 40 g/L glucose, both peptidase and ComX activity decreased almost simultaneously. And although peptidase activity approached zero, the ComX activity appeared to decrease further. This was even more evident when considering the approach with 20 g/L glucose. After *t* = 20 h, ComX activity started to decrease although peptidase activity was already at a low level. This gave reason to investigate the degradation of ComX activity in more detail, with special respect to the influence of extracellular proteases. The highest degradation rate for ComX pheromone of 9.2 ± 2.1 MU/h was found in transition to stationary phase but autodegradation and intracellular degradation caused by the cells appeared to have only a minor influence on the overall degradation rate. Yet, although the modeled EA_CSP_ showed a similar pattern to the calculated degradation rates, the time course did not match the determined overall peptidase activity EA_total_. A simple explanation for this scenario could be, that EA_CSP_ was only partly captured by the applied azocasein assay, as this method is specific for endopeptidase activity. However, ComX also seems to be sensitive towards other proteases, such as metalloproteases (Spacapan et al. 2018), which could not be detected by the applied measurement techniques. It was further noticeable that in all bioreactor processes, surfactin remained stable towards the end of cultivation regardless of the applied glucose concentration. A characteristic decrease of surfactin has however repeatedly been reported (Klausmann et al. 2021; Willenbacher et al. 2015), especially when using high glucose concentrations. Here, this decrease was only observed for shake flask cultivations starting from *t* = 16 h for the approach with 40 g/L glucose and even more prominent when using 20 g/L glucose after *t* = 12 h. Several hypotheses have been proposed to explain this effect. One of the first reasons that comes to mind when only observing the depletion of surfactin could be its use as a nutrient source. This hypothesis was discussed by Klausmann et al. (2021), but immediately refuted, as sufficient amounts of nutrients were still available. In the present study, we also observed that at the end of the shake flask cultivation with 40 g/L glucose about half of the glucose and ammonium source was still available (Additional file 1: Figure S3). In contrast, in the bioreactor cultivation with 40 g/L, glucose was completely consumed, but no depletion of surfactin occurred (Fig. 2). However, the degradation of surfactin during the stationary phase, when the cells are self-maintaining is noticeable in the shake flask cultivation with 40 g/L glucose. When using 8 g/L glucose, the cultivation time is rather short, as a cell decline phase, with the lack of a stationary phase is already observed after 16 h. Increasing glucose concentration leads the cells to enter stationary phase, thus prolonging the cultivation. This is also true for bioreactor cultivations, with the main difference being that the conditions are kept at optimal values with regulated pO_2_ and pH. This leads to the suggestion that during a prolonged stationary phase, cultivation conditions appear to be suboptimal for the cells especially in terms of pH and limited oxygen availability. The pH has a considerable influence on both, surfactin and P_*srfA*_ expression. In 1998, it was already reported by Wei and Chu (1998) that surfactin was no longer present when pH dropped beneath 5.0. In the same year, Cosby et al. (1998) stated that P_*srfA*_ expression is negatively affected below a pH of 5.0, which is especially the case in nutrient rich cultivation media. In addition, possible surfactin precipitation may occur, which is characteristic of a too low pH value around pH 5.0 to 6.0 (Rangarajan and Clarke 2015). Considering these findings, it seems that an increase in glucose concentration is followed by a prolonged cultivation time, which might result in suboptimal conditions in the shake flask, such as too low pH. This may lead to the fact, that the cells have to maintain themselves in the stationary phase under potentially unfavorable conditions, negatively affecting the balance between production to degradation rate as already suggested by Klausmann et al. (2021). Interestingly a similar observation was made for the time course of ComX during shake flask cultivations. Here, a decline was also more prominent with increasing glucose concentration and a stabilizing effect of ComX activity was not evident in the experiment with 40 g/L glucose. This might also be attributed to a shift in the pH, as it has been reported that ComX does not appear to be stable in acidic environments (Okada et al. 2007) and that the isoelectric point for quorum sensing peptides averages 7.1 (Rajput et al. 2015). A more detailed investigation on the influence of the correlation between pH and surfactin or ComX degradation should be targeted in future studies. However, what should be considered for this study is the presumably high sensitivity of ComX towards external factors. Besides the pH, also the oxygen availability has been described as influencing factor (Dogsa et al. 2021). The high sensitivity of ComX can be also reflected in the proposed model, as even small changes in the respective parameters can have a considerable impact on the ComX time course, as illustrated in Fig. 2. This further shows the high complexity of quorum sensing regulated surfactin production and reinforces the need for new and precise process control strategies. Which conditions and more prominently at which time point of the cultivation an optimal ComX concentration could benefit the overall surfactin titer has yet to be explored. However, this might be an interesting approach for future optimization of biotechnological processes using wild-type strains. The proposed model can lay the foundation for a more complex process model for enhanced surfactin production based on quorum sensing and ComX-dependent surfactin expression. The gained knowledge can then be transferred from the model organism *B. subtilis* DSM 10^ T^ to other *Bacillus* strains with special emphasis on agricultural or food applications. With the help of the model presented in this study, such approaches can be realized and applied to enable molecular process control in *Bacillus.*

## Supplementary Information

**Additional file 1: Table S1**. List of strains used for this study.** Table S2**. List of plasmids used for this study.** Table S3**. List of oligonucleotides used for this study according to Hoffmann et al. (2021).** Table S4**. Overview of the specificity test, employing various Bacillus sp.; (+) indicating ComX activity; (-) indicating that no ComX activity above the LOQ (42.7 MU) was determined.** Table S5**. Overview of process parameters of shake flask cultivations, including yield coefficients, highest surfactin titer Pmax [mg/L], highest biomass Xmax [g/L], highest ComX activity ComXmax [MU], as well as the ComX activity at the end of the cultivation ComX tend [MU].** Fig. S1**. (a) Extracted ion chromatogram (XIC) from single ion monitoring (SIM) scan at m/z 681.8747 of cell-free supernatant from B. subtilis DSM 23778; (b) ESI-MS/MS spectrum of the precursor ion m/z 681.8747 at retention time 7.2 min (ComX isomer 1). Identity of the ComX pheromone was confirmed by b- and y-ion series as indicated in blue and red, respectively. Internal fragments are indicated in green. FA: farnesylation. The MS/MS spectrum of ComX isomer 2 at retention time 7.5 min showed almost identical fragment ions (data not shown).** Fig. S2**. Graphical illustration of ComX degradation studies. (a) Plotted are the measured ComX activity (gray bars) and the corresponding ComX concentration determined by mass spectrometry (black circles) over the cultivation time; (b) Plotted are the ComX activity (gray bars) and extracellular peptidase activity (black diamond) of heat-treated supernatant over the incubation time; (c) Plotted are the OD600 (black cross) of strain BKK31700, the corresponding ComX activity (gray bars) and extracellular peptidase activity (black diamond) over the cultivation time.** Fig. S3**. Time course of shake flask cultivations of B. subtilis DSM 10T employing 8, 20 and 40 g/L glucose. Plotted are the CDW (black cross), glucose (gray square) and ammonium (black triangle) depletion against the cultivation time in the upper part and ComX activity (white circle), surfactin concentration (gray circle) and extracellular peptidase activity (black diamond) against the cultivation time in the lower part of the figure.

## Data Availability

All discussed data have been included into the manuscript or the supplementary material. Please turn to the corresponding author for all other requests.

## Abbreviations

CSP: ComX-specific protease
EA: Endopeptidase activity
LOD: Limit of detection
LOQ: Limit of quantification
MSM: Mineral salt medium
MU: Miller units
