# Supplementary material for: Modeling the time course of ComX: towards molecular process control for Bacillus wild-type cultivations
Source: AMB Express. 2021 Oct 29;11:144. doi: 10.1186/s13568-021-01306-5 (PMC8556439; doi:10.1186/s13568-021-01306-5)
Supplement: Supplementary file 1 — Additional file 1: Table S1. List of strains used for this study. Table S2. List of plasmids used for this study. Table S3. List of oligonucleotides used for this study according to Hoffmann et al. (2021). Table S4. Overview of the specificity test, employing various Bacillus sp.; (+) indicating ComX activity; (-) indicating that no ComX activity above the LOQ (42.7 MU) was determined. Table S5. Overview of process parameters of shake flask cultivations, including yield coefficients, highest surfactin titer Pmax [mg/L], highest biomass Xmax [g/L], highest ComX activity ComXmax [MU], as well as the ComX activity at the end of the cultivation ComX tend [MU]. Fig. S1. (a) Extracted ion chromatogram (XIC) from single ion monitoring (SIM) scan at m/z 681.8747 of cell-free supernatant from B. subtilis DSM 23778; (b) ESI-MS/MS spectrum of the precursor ion m/z 681.8747 at retention time 7.2 min (ComX isomer 1). Identity of the ComX pheromone was confirmed by b- and y-ion series as indicated in blue and red, respectively. Internal fragments are indicated in green. FA: farnesylation. The MS/MS spectrum of ComX isomer 2 at retention time 7.5 min showed almost identical fragment ions (data not shown). Fig. S2. Graphical illustration of ComX degradation studies. (a) Plotted are the measured ComX activity (gray bars) and the corresponding ComX concentration determined by mass spectrometry (black circles) over the cultivation time; (b) Plotted are the ComX activity (gray bars) and extracellular peptidase activity (black diamond) of heat-treated supernatant over the incubation time; (c) Plotted are the OD600 (black cross) of strain BKK31700, the corresponding ComX activity (gray bars) and extracellular peptidase activity (black diamond) over the cultivation time. Fig. S3. Time course of shake flask cultivations of B. subtilis DSM 10T employing 8, 20 and 40 g/L glucose. Plotted are the CDW (black cross), glucose (gray square) and ammonium (black triangle) depletion against the cult [file 13568_2021_1306_MOESM1_ESM.pdf]

Supplementary Information

Modeling the time-course of ComX: Molecular process control for *Bacillus* wild-type cultivations

*Chantal Treinen[a], Olivia Magosch[a], Mareen Hoffmann[a], Peter Klausmann[a], Berit Würtz[b], Jens Pfannstiel[b], Kambiz Morabbi Heravi[a], Lars Lilge[a], Rudolf Hausmann[a] and Marius Henkel[a]**

[a]Institute of Food Science and Biotechnology, Department of Bioprocess Engineering (150k), University of Hohenheim, Fruwirthstr. 12, 70599 Stuttgart, Germany

[b]Core Facility Hohenheim, Mass Spectrometry Unit, University of Hohenheim, August-von-Hartmann-Str. 3, 70599 Stuttgart, Germany

***Corresponding author:**	Marius Henkel
Address:	Fruwirthstr. 12, 70599 Stuttgart
Phone:	+49 711 459-24726
Fax:	+49 711 459-24722
Email:	marius.henkel@uni-hohenheim.de

Authors' email addresses:

chantal.treinen@uni-hohenheim.de, olivia.magosch@web.de, hoffmann.mareen@web.de, peterklausmann@gmail.com, berit.wuertz@uni-hohenheim.de, jens.pfannstiel@uni-hohenheim.de, kambiz.morabbi@gmail.com, lars.lilge@uni-hohenheim.de, rudolf.hausmann@uni-hohenheim.de, marius.henkel@uni-hohenheim.de

Table S1 List of strains used for this study

Name	Genotype description and strain designation	Reference
B. subtilis		
DSM 10^T	Wild-type strain (*B. subtilis* Marburg)	DSMZ[a]
DSM 23778	Wild-type strain (*B. subtilis* strain 168)	DSMZ[a]
DSM 1090	Wild-type strain (*B. subtilis* S 237)	DSMZ[a]
JABs24	*trp*+; ∆*manPA*; *sfp*+	(Geissler et al. 2019)[b]
JABs32	*spo0A3*; ∆*manPA*::*erm*; *sfp*+	(Klausmann et al. 2021)[b]
BKK31700	*trpC2*, ∆*comX*::*kan*<br>based on *B. subtilis* strain 168	BGSC[c]<br>(Koo et al. 2017)
CT2	*trpC2*, ∆*comX*::*kan amyE*::[P_{srfA}-*lacZ, spc*]	This study
B. amyloliquefaciens		
DSM 7^T	Wild-type strain (*B. amyloliquefaciens* Fukumoto strain F)	DSMZ[a]
QST 713	Wild-type strain	Serenade® ASO (Bayer AG, Leverkusen, Germany)
B. velezensis		
DSM 23117	Wild-type strain (*B. velezensis* FZB42)	DSMZ[a]
***B.* species**		
DSM 28227	Wild-type strain	DSMZ[a]

[a] Leibniz Institute DSMZ – German Collection of Microorganisms and Cell Cultures GmbH (Braunschweig, Germany)

[b] Kindly obtained from Dr. Josef Altenbuchner, Institute of Industrial Genetics, University of Stuttgart (Stuttgart, Germany)

[c] The Bacillus Genetic Stock Center (Columbus, USA)

Table S2 List of plasmids used for this study

Name	Properties or inserts	Reference
pKAM446	*ori*$_{\text{pUC18}}$, *bla*, *rop*, *ermC*, *amyE′*-[*ter*-P$_{srfAA}$-*lacZ*, *spcR*]- *′amyE*	(Hoffmann et al. 2021)

Table S3 List of oligonucleotides used for this study according to Hoffmann et al. (2021)

Name	Sequence (5' → 3')	Application
S1637	GCGTAATAGACTTTCAGGCGT	Confirmation of P$_{srfA}$-*lacZ* integration into *amyE*
S1638	GCTTCATCCACCACATACAGG	
S1639	AGCCGCTGAAGAATATGG	Sequencing of P$_{srfA}$-*lacZ* integration into *amyE*
S1640	CGTAATGGGATAGGTCAC	

Validation and optimization of ComX pheromone bioactivity assay (*ComX bioassay*)

The reporter strain CT2 was unable to produce its own ComX, which might have biased the results. In this way, ComX-dependent expression of *lacZ* could be measured only when ComX was present in the cell-free supernatant of the sample. To analyze potential background noise, strain CT2 was cultivated and *lacZ* expression was measured directly from the supernatant using the Miller assay (data not shown). An initially high *lacZ* expression could be determined shortly after inoculation, which then decreased with increasing incubation time. Time-turnover curves (data not shown) revealed that an end-point determination was achieved by incubating the mixture for 3 – 5 h respectively. This being also in accordance to Dogsa et al. (2021), who stated a sufficient response 4 h after inoculation. Given a 3 h pre-incubation time of the main culture, total incubation times were 6 – 8 h, which is within the range where background noise is at a minimum. In addition, spectinomycin (100 µg/mL) was added to the main culture. In this way, a sterile filtration step of the sample was not necessary, since the strains under study cannot grow on spectinomycin, thus saving time and resources.

Specificity and proof-of-concept of ComX bioassay

The activity of the ComX pheromone is specific for individual *Bacillus* spp. (Ansaldi et al. 2002). To validate that the assay was specific for *B. subtilis* strains used in our laboratory, various *Bacillus* sp. and *B. subtilis* subsp. were cultivated in biological duplicates. The main culture was carried out in baffled shake flasks with relative filling volumes of 0.1 mL/mL (10%) MSM with 40 g/L glucose and a starting OD_{600} of 0.05, as cultures were inoculated at night to allow sampling on the next day. Samples were taken regularly and tested for ComX activity using the bioassay. Strain BKK31700, which was not able to produce ComX was used as negative control. All strains were tested at t_0 as an additional negative control, showing no signal above the respective LOQ (data not shown). As expected, a ComX activity could not be obtained for the negative control or *Bacillus* species other than *B. subtilis* (Table S4). Sequence comparison of strain DSM 10^T (NCBI: CP060710.1) (Lilge et al. 2021) and strain 168 (NCBI: NC_000964.3) revealed the same ComX peptide sequence (ADPITRQWGD) for both strains, indicating similar pheromone structure. For liquid chromatography-mass spectrometry (LC-MS), shake flask cultivations were carried out as previously described with a starting OD_{600} of 0.1. Samples for offline measurement were withdrawn after 14 h of cultivation at the approximate peak of ComX activity with 252.7 MU. Cell-free supernatant from *B. subtilis* DSM 23778 was examined by the ComX bioassay and LC-MS/MS. The results of the LC-MS/MS analyses are shown in Figure S1a as extracted ion chromatogram (XIC) from a single ion monitoring (SIM) scan of the ComX pheromone $[M + 2H + farnesyl (W)]^{2+}$ = 681.8747 *m*/*z*. Peaks 1 and 2 at retention times of 7.2 min and 7.5 min correspond probably to different structural isomers of ComX. Identity of the ComX pheromone was confirmed by MS/MS spectra (Figure S1b).

Table S4 Overview of the specificity test, employing various *Bacillus* sp.; (+) indicating ComX activity; (-) indicating that no ComX activity above the LOQ (42.7 MU) was determined

**Strain**	**ComX activity**
Negative control	
BKK31700	–
B. subtilis subsp.	
DSM 10^{T}	+
DSM 23778	+
DSM 1090	+
JABs24	+
JABs32	+
Bacillus spp.	
B. amyloliquefaciens DSM 7^{T}	–
B. amyloliquefaciens QST 713	–
B. velezensis DSM 23117 (FZB42)	–
DSM 28227	–

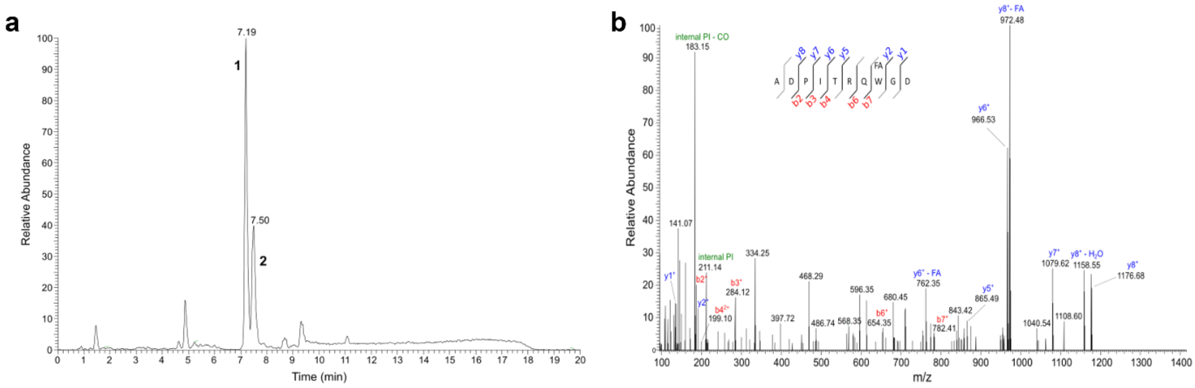

Fig. S1 (a) Extracted ion chromatogram (XIC) from single ion monitoring (SIM) scan at *m/z* 681.8747 of cell-free supernatant from *B. subtilis* DSM 23778; (b) ESI-MS/MS spectrum of the precursor ion *m/z* 681.8747 at retention time 7.2 min (ComX isomer 1). Identity of the ComX pheromone was confirmed by b- and y-ion series as indicated in blue and red, respectively. Internal fragments are indicated in green. FA: farnesylation. The MS/MS spectrum of ComX isomer 2 at retention time 7.5 min showed almost identical fragment ions (data not shown)

a

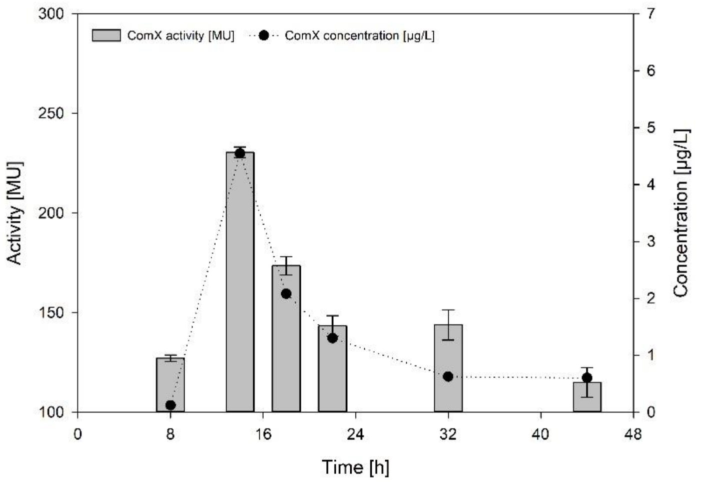

b

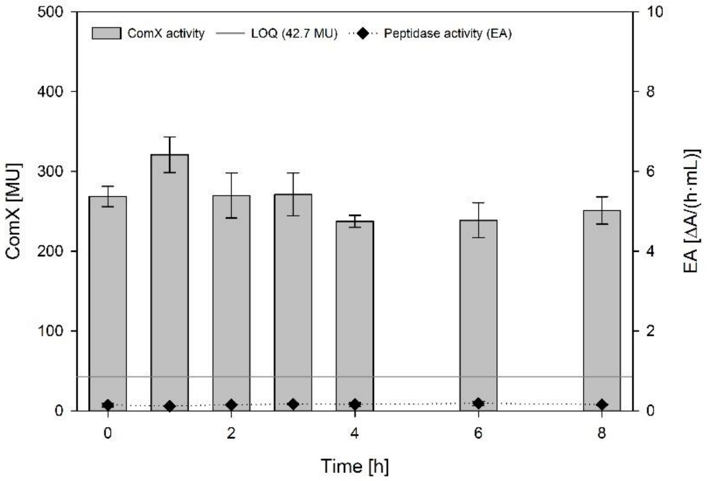

c

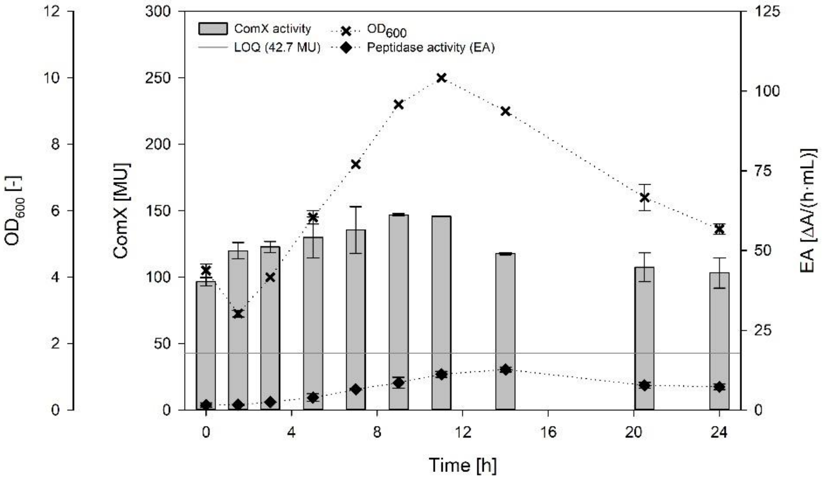

Fig. S2 Graphical illustration of ComX degradation studies. (a) Plotted are the measured ComX activity (gray bars) and the corresponding ComX concentration determined by mass spectrometry (black circles) over the cultivation time; (b) Plotted are the ComX activity (gray bars) and extracellular peptidase activity (black diamond) of heat-treated supernatant over the incubation time; (c) Plotted are the OD_{600} (black cross) of strain BKK31700, the corresponding ComX activity (gray bars) and extracellular peptidase activity (black diamond) over the cultivation time

Time-course of ComX pheromone activity during shake flask cultivation

To relate the time-course of ComX pheromone activity to surfactin formation, natural surfactin-forming wild-type strain *B. subtilis* DSM 10^T was first cultivated in shake flasks. For this purpose, varying glucose concentrations were used to depict different lengths of growth phases and glucose consumption patterns. The results of the cultivations are summarized in Figure S2, as well as Table S5. The lowest applied glucose concentration was 8 g/L, as recommended by Willenbacher et al. (2015). The CDW reached its maximum of 2.6 ± 0.1 g/L after 16 h of cultivation and decreased as soon as glucose was depleted without entering a stationary phase. There was no decrease in surfactin; in fact, P_{max} of 323.0 ± 36.8 mg/L was reached toward the end of cultivation. After CDW_{max} was reached, peptidase activity decreased slightly and stayed consistent at around 23 – 25 ΔA/(mL·h). In comparison, a reverse pattern was observed for shake flask cultivation with 40 g/L glucose. The CDW did not decrease after 16 h but entered the stationary phase with 18.8 ± 0.9 g/L glucose and 0.9 ± 0.1 g/L ammonium remaining after 40 h of cultivation. Surfactin, however, reached the maximum between 12 – 16 h of cultivation and decreased afterwards almost toward zero. Thereby, a P_{max} of 389.6 ± 12.9 mg/L was detectable after 12 h. After reaching its peak of 45.9 ± 3.4 ΔA/(mL·h) at t = 16 h, peptidase activity went almost to zero. Since glucose was not completely consumed in the approach with 40 g/L glucose, it was omitted to test higher glucose concentrations. In a further experiment, 20 g/L glucose were employed, where an intermediate effect could be detected. Similar to 40 g/L glucose, there was a prolonged stationary phase after CDW_{max} of 5.9 ± 0.1 g/L was reached at t = 24 h, but in this case the glucose was completely consumed during cultivation. In addition, a surfactin peak of P_{max} = 451.5 ± 36.4 mg/L was reached after 12 h, after which a rapid degradation occurred. A similar behavior was observed for peptidase activity. Here, a peak value of 34.7 ± 3.1 ΔA/(mL·h) was observed at t = 12

h, after which the activity decreased in the direction of zero. Considering the behavior of ComX, a comparable trend was visible in all cultivations. An increase in ComX activity was observed simultaneously with an increase in biomass during the exponential growth phase. The highest ComX activity was measured between 12 – 20 h, which broadly corresponded to the transition phase. For the cultivation with 8 g/L glucose, a maximum value at 278.9 ± 2.9 MU was detected after 20 h, having a similar highpoint at 276.9 ± 2.4 MU after 12 h. For 20 g/L glucose the highpoint at 289.9 ± 21.5 MU was measured after 16 h, and for 40 g/L glucose at 357.3 ± 41.9 MU after 12 h. With entry into stationary phase, there was a decrease in ComX activity, which was more pronounced with increasing glucose concentration. In case of 8 g/L glucose, ComX activity decreased slightly and remained consistent at around 190 – 200 MU. For the cultivation with 20 g/L glucose an extended degradation phase of ComX activity was observed, stagnating around 140 – 150 MU. A continuous downward trend was noticed for the cultivation using 40 g/L glucose with only 93.1 ± 1.6 MU remaining after 40 h of cultivation.

Table S5 Overview of process parameters of shake flask cultivations, including yield coefficients, highest surfactin titer P_{max} [mg/L], highest biomass X_{max} [g/L], highest ComX activity $ComX_{max}$ [MU], as well as the ComX activity at the end of the cultivation ComX t_{end} [MU]

Parameter \ Cultivation	Shake flask cultivation					
	**8 g/L**		**20 g/L**		**40 g/L**	
**X_{max}** [g/L]	2.6 ± 0.1	16 h	5.9 ± 0.1	24 h	5.4 ± 0.3	32 h
**P_{max}** [mg/L]	323.0 ± 36.8	40 h	451.5 ± 36.4	12 h	389.6 ± 12.9	12 h
**ComX**$_{max}$ [MU]	278.9 ± 2.9	20 h	289.9 ± 21.5	16 h	357.3 ± 41.9	12 h
**ComX** [MU] t_{end}	204.2 ± 4.4	40 h	146.5 ± 15.1	40 h	93.1 ± 13.6	40 h
$Y_{X/S}$ [g/g] at $X_{\geq 90\%}$	0.25		0.26		0.17	
$Y_{P/S}$ [g/g] at $P_{\geq 90\%}$	0.03		0.05		0.03	
$Y_{P/X}$ [g/g] at $P_{\geq 90\%}/X_{\geq 90\%}$	0.19		0.17		0.17	
μ_{max} [1/h]	0.68		0.63		0.54	
$q_{overall}$ [g/(g·h)]	0.02		0.01		0.01	

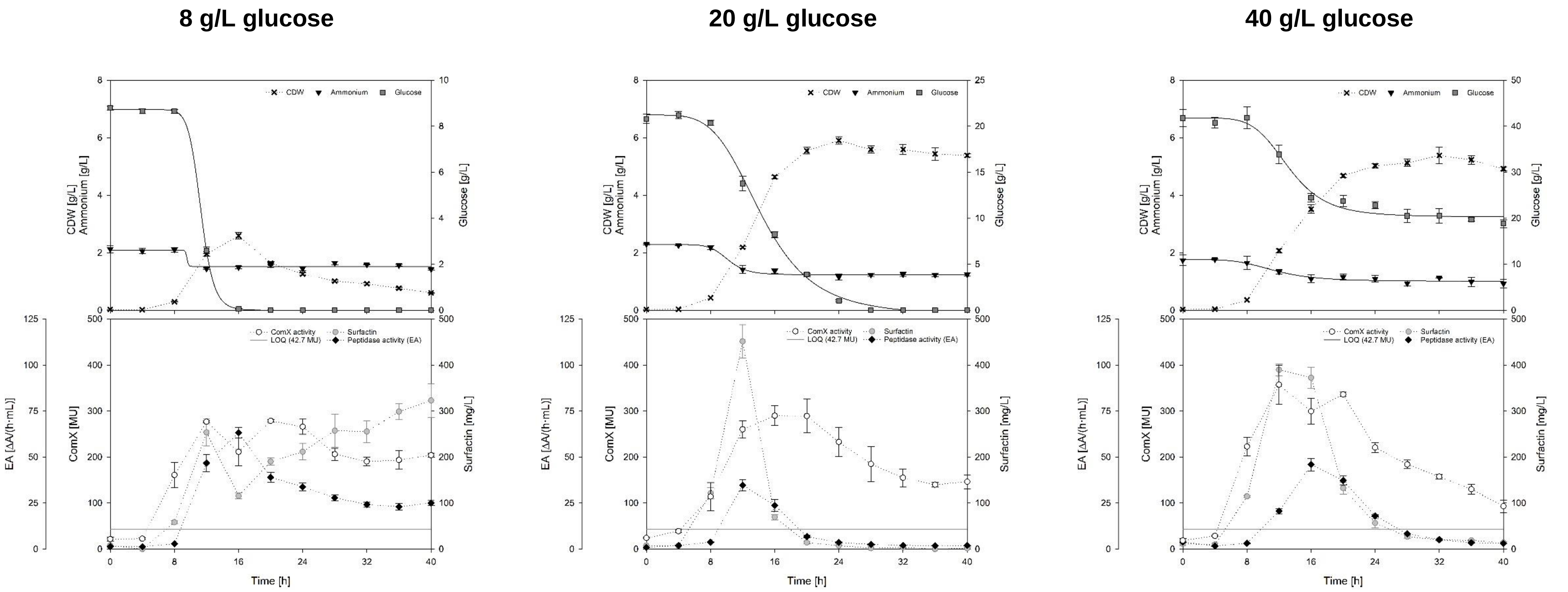

Fig. S3 Time course of shake flask cultivations of *B. subtilis* DSM 10^T employing 8, 20 and 40 g/L glucose. Plotted are the CDW (black cross), glucose (gray square) and ammonium (black triangle) depletion against the cultivation time in the upper part and ComX activity (white circle), surfactin concentration (gray circle) and extracellular peptidase activity (black diamond) against the cultivation time in the lower part of the figure

References

Ansaldi M, Marolt D, Stebe T, Mandic-Mulec I, Dubnau D (2002) Specific activation of the *Bacillus* quorum-sensing systems by isoprenylated pheromone variants. Mol Microbiol 44:1561–1573.

Dogsa I, Spacapan M, Dragoš A, Danevčič T, Pandur Ž, Mandic-Mulec I (2021) Peptide signaling without feedback in signal production operates as a true quorum sensing communication system in *Bacillus subtilis*. Commun Biol 4:58.

Geissler M, Kühle I, Morabbi Heravi K, Altenbuchner J, Henkel M, Hausmann R (2019) Evaluation of surfactin synthesis in a genome reduced *Bacillus subtilis* strain. AMB Expr 9:84.

Hoffmann M, Braig A, Fernandez Cano Luna DS, Rief K, Becker P, Treinen C, Klausmann P, Morabbi Heravi K, Henkel M, Lilge L, Hausmann R (2021) Evaluation of an oxygen-dependent self-inducible surfactin synthesis in *B. subtilis* by substitution of native promoter P_{srfA} by anaerobically active P_{narG} and P_{nasD}. AMB Expr 11:57.

Klausmann P, Hennemann K, Hoffmann M, Treinen C, Aschern M, Lilge L, Morabbi Heravi K, Henkel M, Hausmann R (2021) *Bacillus subtilis* high cell density fermentation using a sporulation-deficient strain for the production of surfactin. Appl Microbiol Biotechnol 105:4141–4151.

Koo BM, Kritikos G, Farelli JD, Todor H, Tong K, Kimsey H, Wapinski I, Galardini M, Cabal A, Peters JM, Hachmann A-B, Rudner DZ, Allen KN, Typas A, Gross CA (2017) Construction and analysis of two genome-scale deletion libraries for *Bacillus subtilis*. Cell Syst 4:291-305.

Lilge L, Hertel R, Morabbi Heravi K, Henkel M, Commichau FM, Hausmann R (2021) Draft genome sequence of the type strain *Bacillus subtilis* subsp. *subtilis* DSM10. Microbiol Resour Announc 10:e00158-21.

Willenbacher J, Yeremchuk W, Mohr T, Syldatk C, Hausmann R (2015) Enhancement of surfactin yield by improving the medium composition and fermentation process. AMB Expr 5:57.
